# Comparative Integrated Omics Analysis of the Hfq Regulon in *Bordetella pertussis*

**DOI:** 10.3390/ijms20123073

**Published:** 2019-06-24

**Authors:** Ana Dienstbier, Fabian Amman, Daniel Štipl, Denisa Petráčková, Branislav Večerek

**Affiliations:** 1 Laboratory of post-transcriptional control of gene expression, Institute of Microbiology v.v.i., 14220 Prague, Czech Republic; ana.sencilo@biomed.cas.cz (A.D.); daniel.stipl@biomed.cas.cz (D.S.); petrack@biomed.cas.cz (D.P.); 2 Institute for Theoretical Chemistry, University of Vienna, Währinger Straße 17, A-1090 Vienna, Austria; fabian@tbi.univie.ac.at; 3 Division of Cell and Developmental Biology, Medical University of Vienna, Schwarzspanierstraße 17, A-1090 Vienna, Austria

**Keywords:** *Bordetella pertussis*, Hfq, omics analysis, T3SS, serum resistance, solute-binding proteins

## Abstract

*Bordetella pertussis* is a Gram-negative strictly human pathogen of the respiratory tract and the etiological agent of whooping cough (pertussis). Previously, we have shown that RNA chaperone Hfq is required for virulence of *B. pertussis*. Furthermore, microarray analysis revealed that a large number of genes are affected by the lack of Hfq. This study represents the first attempt to characterize the Hfq regulon in bacterial pathogen using an integrative omics approach. Gene expression profiles were analyzed by RNA-seq and protein amounts in cell-associated and cell-free fractions were determined by LC-MS/MS technique. Comparative analysis of transcriptomic and proteomic data revealed solid correlation (r^2^ = 0.4) considering the role of Hfq in post-transcriptional control of gene expression. Importantly, our study confirms and further enlightens the role of Hfq in pathogenicity of *B. pertussis* as it shows that Δ*hfq* strain displays strongly impaired secretion of substrates of Type III secretion system (T3SS) and substantially reduced resistance to serum killing. On the other hand, significantly increased production of proteins implicated in transport of important metabolites and essential nutrients observed in the mutant seems to compensate for the physiological defect introduced by the deletion of the *hfq* gene.

## 1. Introduction

*Bordetella pertussis* is a Gram-negative strictly human pathogen of the respiratory tract and the etiological agent of whooping cough (pertussis) [1]. This highly contagious disease is especially severe in infants and remains a major cause of infant mortality and morbidity worldwide, predominantly in developing countries [2]. Furthermore, pertussis incidence is currently on the rise in industrialized countries with highly vaccinated populations [3,4]. While there are several reasons for this phenomenon [5], there are two major disease-related factors contributing to recent increase in pertussis cases: short-lived immunity induced by current acellular vaccines and pathogen adaptation leading to escape from the immunity by antigenic variation [6,7,8]. The global reemergence of pertussis clearly suggests that we need to widen our understanding of the molecular mechanisms underlying the pathogenesis of *B. pertussis* [9,10]. In many pathogenic bacteria the RNA chaperone Hfq and small non-coding regulatory RNAs (sRNAs) emerged as critical players in posttranscriptional regulation of virulence and physiological fitness [11,12,13]. The Hfq protein forms ring-shaped hexamers that possess several RNA binding sites that allow for simultaneous interaction with both sRNA and mRNA molecules and stabilization of their interactions [14,15,16]. Besides its role in facilitation and stabilization of RNA duplexes, Hfq can actively remodel the structure of RNAs and also increase the stability of sRNAs [14,16,17].

Recently we have shown that Hfq is required for virulence of *B. pertussis* as the Δ*hfq* mutant was affected both in its ability to efficiently multiply and persist in mouse lungs as well as in its capacity to cause a lethal infection in mouse [18]. Furthermore, our global DNA microarray-based transcriptomic profiling of the *hfq* mutant suggested that Hfq protein significantly affects expression of more than 10% annotated genes [19]. Nevertheless, despite the high sensitivity, transcriptomic profiling does not capture post-transcriptional and post-translational modifications that affect the amounts of produced proteins. On the other hand, mass spectrometry-based proteomics lacks the sensitivity to detect low abundant proteins. Therefore, integrative analysis of both transcriptomic and proteomic datasets enables a more complete understanding of studied biological processes [20,21]. First studies based on such an approach revealed that the overlap between the outcomes of transcriptomic and proteomic analyses is not extensive irrespective of the organism [22,23,24]. This discrepancy was attributed in part to technological limitations of applied procedures and in part to inherent biological complexity of transcription and translation processes [25,26]. Especially, factors linked to translational efficiency, such as codon usage bias, strength and accessibility of ribosome binding site, secondary structure and stability of the transcript, and post-transcriptional activity of the regulatory proteins, contribute to poor correlation between determined transcript and protein levels [20,27,28,29]

Hfq is a key player in post-transcriptional control of gene expression in Gram-negative bacteria and therefore, its biological activities should in principle weaken the correlation between the gene expression and protein synthesis profiles. Recently, an integrative analysis of Hfq-specific transcriptomic and proteomic profiles based on high-throughput RNA-seq and LC-MS/MS technologies was performed in soil bacterium *Pseudomonas fluorescence*. It showed that such a multiomics approach allows for dissection of discrete contributions of Hfq to gene regulation at different levels [30]. Therefore, we were interested to perform such a comparative analysis in human pathogen to elucidate the Hfq-related variations at both transcriptomic and proteomic levels per se and also to decipher how the changes in gene expression profiles translate into protein production in *B. pertussis*. Our results indicate that considering the role of Hfq in the post-transcriptional control of gene expression, the correlation between transcriptome and proteome is relatively high. Furthermore, our data corroborate and further clarify the necessity of Hfq for physiological fitness and pathogenicity of *B. pertussis*.

## 2. Results

### 2.1. Identification of the Hfq Regulon by RNA-seq

**Figure 1:**
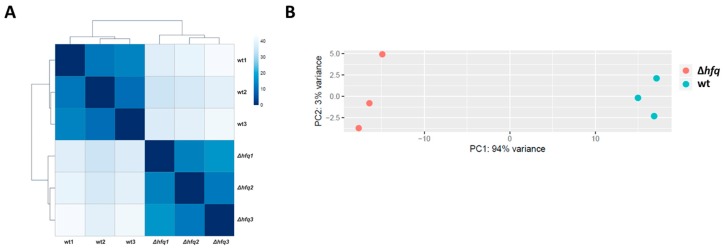
Clustering of transcriptomic data. (**A**) Heat map showing hierarchical clustering of the Euclidean sample-to-sample distance between transcriptomic profiles of wt and Δ*hfq* mutant. (**B**) Principal component analysis was applied to transcriptomic profiles of the wt strain (blue circles) and Δ*hfq* mutant (red circles). Each dot represents an independent biological replicate.

Samples of total RNA isolated from biological triplicates of *B. pertussis* Tohama I strain and its isogenic Δ*hfq* strain cultures were analyzed by RNA-seq. RNA-seq analysis yielded on average 16 million reads, which were mapped to the *B. pertussis* genome. The comparison of global expression profiles showed that biological replicates of either wt or Δ*hfq* cells are highly uniform and thereby reproducible (Figure 1A). Principal component analysis (PCA) revealed that samples from wt strain and *hfq* mutant clustered separately along principal component 1 (94%) reflecting global changes in gene expression profiles resulting from deletion of the *hfq* gene (Figure 1B).

Differential expression (DE) analysis identified 653 significantly modulated *B. pertussis* genes (|log_2_FC| > 1; *q* < 0.05) including 40 non-coding RNAs and 11 transfer RNA genes (Table S1). Among the DE genes, 281 genes were downregulated and 372 upregulated in the Δ*hfq* strain. Remarkably, 56 genes (8.3% of all DE genes) encoding the components of ATP-binding cassette transport system were significantly up- or downregulated in the mutant. In agreement with our previous microarray study, the expression of several genes within the type III secretion (T3SS) *bcs*/*btr* locus including *bsp22*, *bopN*, *bopB*, and *bopD* was substantially decreased in the mutant. Genes involved in iron–sulfur cluster protein biogenesis (*iscU*, *iscA, iscS*) were also highly downregulated in the *hfq* mutant. Among the genes displaying increased expression in the *hfq* mutant were those coding for ribosomal proteins, amino acid biosynthesis and transport, and, surprisingly, genes encoding pertussis toxin subunits and its secretion apparatus (*ptx*/*ptl* locus).

**Figure 2:**
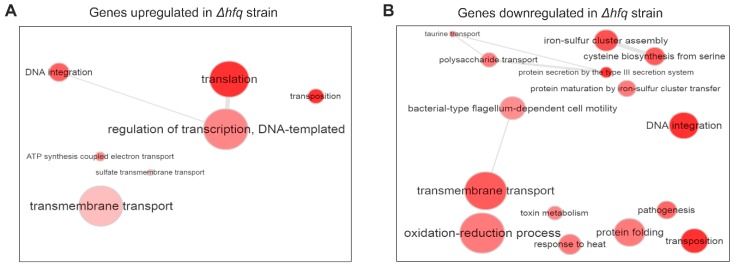
Gene ontology (GO) term analysis of genes significantly downregulated (**A**) or upregulated (**B**) in the Δ*hfq* mutant. Significantly enriched terms from the domain ‘Biological processes’ and their catenations are summarized and visualized by REVIGO as an interactive graph. Circle size encodes number of genes associated with respective category and red shades encode the significance level of the enrichment.

To get better insight into the functional profiles of Hfq-dependent genes we performed gene ontology (GO) enrichment analysis using all DE genes. GO term analysis revealed that genes belonging to several biological processes were significantly enriched in both up- and downregulated gene sets. As shown in Figure 2A, within the set of genes which were significantly upregulated in the *hfq* mutant, categories such as “Translation”, “Regulation of transcription”, and “Transmembrane transport” were highly enriched. On the other side, genes belonging to “Transmembrane transport”, “Iron–sulfur cluster assembly”, “Oxido-reduction process”, “Pathogenesis”, and “Protein secretion by the type III secretion system” terms were enriched among the transcripts which were significantly downregulated in the *hfq* mutant (Figure 2B). Apparently, GO term analysis of the DE genes recapitulated many of the observations from our previous studies [19].

### 2.2. The Effect of Hfq on Proteome and Secretome Composition of B. pertussis

**Figure 3:**
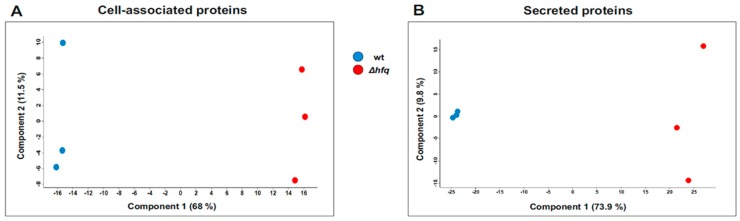
Principal component analysis (PCA) of proteomic samples. PCA was applied to protein profiles of the wt strain (blue circles) and Δ*hfq* mutant (red circles) determined in corresponding cell-associated (**A**) or secreted (**B**) protein fractions. Each dot represents an independent biological replicate.

LC-MS/MS analysis of the *B. pertussis* proteome and secretome identified 1631 and 733 proteins, respectively, whose label-free quantification (LFQ) intensities passed our detection criteria. Principal component analysis of both datasets revealed that protein profiles identified in samples of the wt strain clustered separately from those of the Δ*hfq* strain (Figure 3).

**Figure 4:**
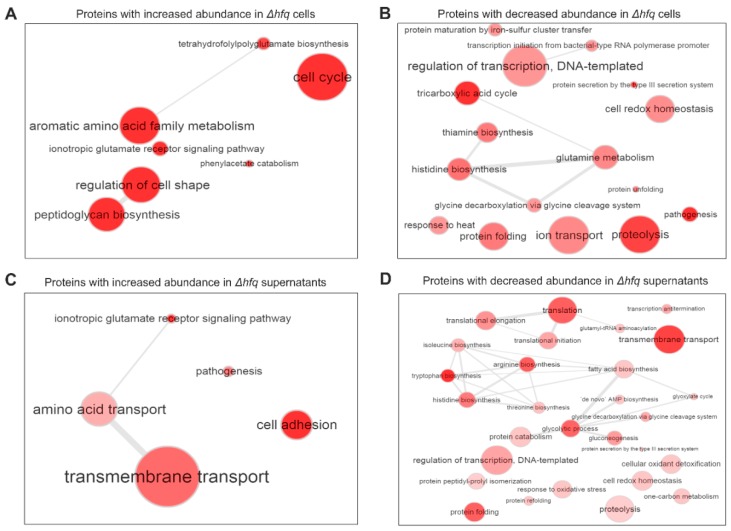
GO term enrichment analysis of genes either significantly upregulated (**A**,**C**) or downregulated (**B**,**D**) in the Δ*hfq* cells and Δ*hfq* culture supernatants, respectively. Biological processes significantly enriched for genes belonging to corresponding functional category are shown as an interactive graph. Significantly enriched terms from the domain “Biological processes” and their catenations are summarized and visualized by REVIGO. Circle size encodes number of genes associated with respective categories, red shades encode the significance level of the enrichment.

For pellet proteins, the production of 489 proteins was found to be significantly modulated between wt and *hfq* mutant strains (Table S2). The abundance of 219 proteins was higher in the mutant including 19 “ON” proteins which were not detected in the wt strain. Among this set of proteins, GO terms such as “Cell cycle”, “Peptidoglycan synthesis”, and “Aromatic amino acid metabolism” were significantly enriched (Figure 4A). On the other hand, 270 proteins displayed significantly higher LFQ intensities in the wt strain including 10 “OFF” proteins which, in contrast to the wt samples, were not detected in the mutant. GO term analysis revealed that biological processes such as “Proteolysis”, “Ion transport”, “Pathogenesis”, and “Protein secretion by the type III secretion system” were enriched among this group of proteins (Figure 4B).

As for the secreted fraction (Table S3), abundance of 114 proteins was higher in the mutant (including six “ON” proteins) and these proteins clustered into categories such as “Transmembrane transport”, “Cell adhesion”, and “Amino acid transport” (Figure 4C). On the other hand, 445 proteins displayed significantly higher LFQ intensities in the wt strain (including 136 “OFF” proteins). The GO term analysis of these differentially secreted proteins identified rather broad variety of processes including “Transmembrane transport”, “Proteolysis”, “Response to oxidative stress”, “Protein secretion by the type III secretion system”, and several processes linked to translation and amino acid biosynthesis (Figure 4D).

Among the proteins with highly increased abundance in the *hfq* mutant in both proteome and secretome datasets were prevalently members of various transporters including tripartite tricarboxylate transporters (TTT) (BP2066, BP3501, and BP1358), ABC transporters (BP2090, BP2352, and BP0663) and tripartite ATP-independent periplasmic transporters [31] (BP1487 and BP1489), lipoprotein BP2271, adhesin FhaS and all five pertussis toxin subunits. Of note, genes encoding these overproduced proteins also belonged to the set of the most upregulated genes in the mutant strain (Table 1).

A substantial number of proteins displayed significantly diminished levels in the *hfq* mutant in both proteomic analyses. The largest group of such proteins belonged to T3SS structural components and its secreted substrates. Among the 20 proteins displaying the most decreased abundance in the Δ*hfq* cells were nine T3SS-specific proteins. Likewise, in the supernatant fraction, amounts of Bsp22 and BopD proteins were dramatically reduced (log_2_FC < −9) while BopC, Bcr4, BopB and BopN proteins could not be detected (Table 2).

**Figure 5:**
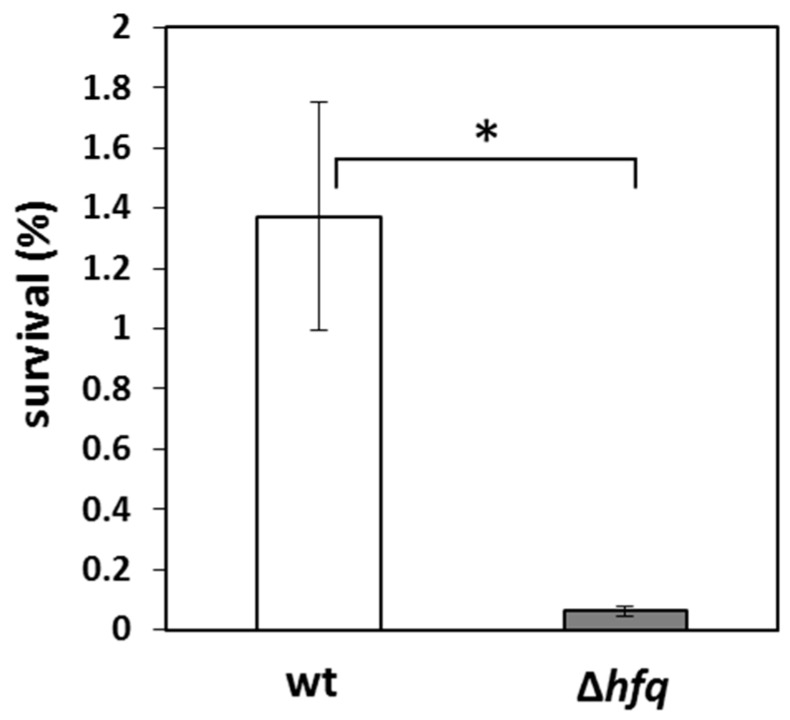
Serum killing assay of Δ*hfq* and Tohama I strains. Serum resistance is expressed as percentage of wt (white bar) and Δ*hfq* (grey bar) cells that survived upon incubation with 10% human serum when compared to controls (bacteria incubated with heat-inactivated serum). The error bars represent the standard deviation of the mean obtained from three biological replicates (*, *p* ˂ 0.001). The result is representative of three independent experiments.

Noticeably, one of the virulence factors displaying consistently reduced expression, production and secretion in the *hfq* mutant was the autotransporter Vag8. The Vag8 protein binds and recruits C1 esterase inhibitor and thereby inhibits complement activity and contributes to serum resistance [32,33,34]. Therefore, we asked whether the reduced production of Vag8 factor would compromise the capacity of the Δ*hfq* strain to evade complement-mediated killing. When compared to recent isolates, Tohama I strain exhibits high susceptibility to serum killing [34], nevertheless, in line with our assumption, the survival of the *hfq* mutant (0.06% ± 0.01%) was dramatically decreased when compared to the wt strain (1.36% ± 0.38%) (Figure 5).

### 2.3. Correlation between Transcriptome, Proteome and Secretome Datasets

**Figure 6:**
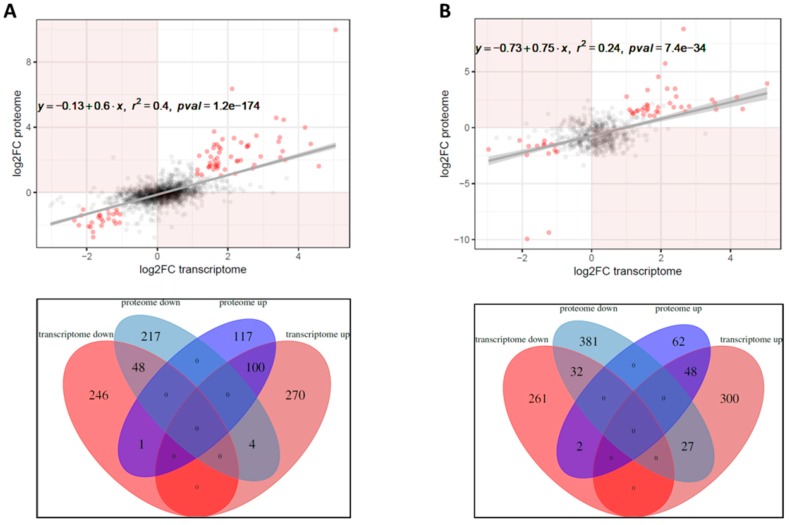
Correlation analysis between transcriptomic and proteomic datasets. (**A**) Scatterplots representing the pairwise comparisons of Δ*hfq*/wt log_2_ ratios between transcriptome and either proteome (left) or secretome (right). Only the genes for which levels of corresponding proteins were reliably detected by label-free quantification were used for correlation analysis. Red dots depict genes which are significantly deregulated in both datasets (*p*-value < 0.05, |log_2_FC| > 1). Line depicts the best fit as predicted by linear regression. (**B**) Venn diagrams showing the number of differentially expressed genes and proteins and the overlap between each dataset. Left: comparison of transcriptome and proteome. Right: comparison of transcriptome and secretome. Zero values indicate intersections which can not materialize (i.e., being up- and downregulated in the same dataset).

Considering previous reports, the correlation between RNA-seq and LC-MS/MS analysis of cell-associated proteins was relatively high (r^2^ = 0.40, *p*-value 1.2 × 10^−174^) with 148 proteins displaying same trend in abundance as corresponding genes in transcriptomic profiling (Figure 6A). As shown in Figure 6B, the concordance of RNA-seq data with secretome analysis was much less positive (r^2^ = 0.24, *p*-value 7.4 × 10^−34^) with only 80 proteins showing similar trend between both analyses. Of note, among genes showing strong correlation with both proteomic datasets were those encoding the T3SS apparatus, pertussis toxin and its transport machinery, ABC, TRAP, and TTT transporters and other proteins involved in the primary metabolism. Apparently, highly modulated genes showed better correlation with abundance of corresponding proteins than those for which the expression was changed only slightly above the thresholds of significance.

## 3. Discussion

In this study we present first integrative omics analysis of the Hfq regulon in the human pathogen. Our study had several objectives: (a) to corroborate the outcomes of our previous transcriptomic study, (b) identify novel targets of Hfq-specific regulatory activities using high-throughput omics techniques, and (c) compare and evaluate the general effects caused by an important post-transcriptional regulator at the level of transcriptome, proteome, and secretome. Compared to microarray profiling (368 protein coding differentially expressed (DE) genes), the differential expression RNA sequencing identified almost two-fold higher number of deregulated genes (602 protein coding DE genes) in the *hfq* mutant. This finding is not surprising considering the higher sensitivity and reproducibility of the RNA-seq method compared to DNA microarray technique [35,36].

Our data are in line with previous studies reporting modest correlation between transcriptomic and proteomic analyses. Nevertheless, considering the role of Hfq in the post-transcriptional control of gene expression, the correlation coefficient between transcriptome and proteome is relatively high when compared to other studies [22,23,37]. The comparison of RNA-seq with secretome analysis output yielded lower correlation values. This finding can possibly result from “contamination” of bacterial culture supernatants with abundant cytosolic proteins such as components of transcriptional and translational machineries. We speculate that these proteins were released from lysed cells during cultivation and sample preparation and therefore their levels do not correspond to changes in gene expression profiles between wt and Δ*hfq* strains.

Importantly, we corroborated several Hfq-specific effects on gene expression profiles which were seen in our previous microarray study [19]. We recapitulated the strong requirement of Hfq chaperone for T3SS functionality as the expression of T3SS genes and production as well as secretion of T3SS components were significantly reduced in the *hfq* mutant. Especially the differences observed in culture supernatants were enormous (more than two orders of magnitude). Several regulators were shown to play a role in control of T3SS activity in *B. pertussis*, including BvgAS two-component system and BtrAS regulatory circuit [38,39]. The response regulator BvgA activates the expression of an extracytoplasmic function sigma factor *btrS* (BP2234) as well as of *btrU*, *btrV*, and *btrW* genes [40]. While BtrS was shown to be required for efficient transcription of the *bsc* locus encoding the T3SS injectisome, BtrU, BtrV, and BtrW regulatory proteins encoded within the *btr* locus are required for secretion through the T3SS apparatus. Recently, a secreted antagonist of BtrS factor called BtrA (BP2233) exerting negative control over the expression of *Bordetella* T3SS genes was reported [39,41]. Secretion of the BtrA inhibitor reactivates BtrS and, consequently, activates the expression of the T3SS genes [39]. Nevertheless, we did not observe any significant changes in expression of the *btrAS* regulatory node. Moreover, BtrA protein could be detected only in the pellets and its levels were decreased in the mutant (log_2_FC of −0.53). Apparently, the reduced expression, production and in particular secretion of T3SS components observed in the *hfq* mutant are independent of *btrAS* circuit. Relatively high LFQ intensities of T3SS secreted substrates detected in the wt strain were rather surprising. *B. pertussis* Tohama I represents a laboratory-adapted strain and was suggested to lose its ability to secrete T3SS components during long-term in vitro passaging [40,42,43]. Nevertheless, the capacity to secrete T3SS substrates in Tohama I can be regained upon contact with the host [19,43] or under nutrient limitation [44,45]. We did not use iron- or glutamate-limited media in our experiments and cells were collected in mid exponential phase of growth. Nevertheless, we cannot completely rule out the possibility that our cultures were partially nutrient-limited at the time of harvest. Of note, when compared to Hfq-specific effects at transcriptional and translational levels, the massive differences in protein abundances seen in culture supernatants suggest that Hfq is indirectly required for efficient secretion process through the T3SS apparatus.

In line with our previous reports, expression and production of autotransporter Vag8, a major player in complement evasion [32,33,34], was significantly reduced in the *hfq* mutant. In support, the *hfq* mutant displayed strongly reduced resistance to serum killing. Increased serum sensitivity of the *hfq* mutant was described also in *Neisseria meningitidis* [46]. We assume that this phenotype can be ascribed to reduced production of the Vag8 protein, as the amounts of BrkA, FhaB, and BapC factors reported to be involved in diversion of complement-mediated killing [47,48,49] were comparable (BrkA) or even higher in the mutant (FhaB). BapC autotransporter was not detected by proteomics and expression of *bapC* gene was increased in the mutant.

Similarly to several other *hfq*-deficient bacteria, the Δ*hfq* strain of *B. pertussis* displays growth deficit. Based on the results of our microarray study we hypothesized that *hfq* mutant of *B. pertussis* compensates the slower growth with increased production of translation machinery components and proteins involved in transport of nutrients [19]. In support, our current study reveals that the most upregulated genes and corresponding proteins found in the *hfq* mutant are represented predominantly by different types of transport proteins, namely, TTT, TRAP, and ABC transporter families. These solute-binding protein-dependent transporters allow uptake even at very low concentrations of ligands [50]. Interestingly, TTT family transporter genes called “Bug” genes (Bordetella uptake genes) are highly overrepresented in the *B. pertussis* genome as they encode 81 functional TTT proteins [51]. While ligands for majority of these proteins are unknown, crystal structures of BugD and BugE proteins identified their ligands as aspartate and glutamate, respectively [52,53]. Intriguingly, expression of *ptx*/*ptl* locus and, consequently, production and secretion of pertussis toxin subunits was significantly increased in the *hfq* mutant. In the light of reduced virulence of the mutant and the importance of this toxin for *B. pertussis* pathogenicity [54] it is rather surprising observation which may be conceived as compensatory response to the lack of Hfq.

With regard to observed high impact of *hfq* deletion on gene expression profiles it is of particular interest that abundance of at least 16 transcriptional regulators and five alternative sigma factors was significantly modulated in the *hfq* mutant. These results are in line with already described roles of Hfq in expression of alternative sigma factors [55,56,57,58] and suggest that similarly to other bacteria, a substantial part of the Hfq-specific effects seen in *B. pertussis* represents indirect regulation. For example, the expression and production of the iron transport repressor Fur is increased in the *hfq* mutant of *B. pertussis* and, consequently, the expression of several genes responsible for iron delivery was decreased in the mutant. One of the surprising results of this study was the relatively low impact of Hfq on abundance of non-coding RNAs. Recently we have identified small non-coding RNA RgtA that is involved in the regulation of the transport of glutamate, a key metabolite in the *B. pertussis* physiology and the abundance of which in the *hfq* mutant was strongly reduced [59]. Nevertheless, our data indicate that only 40 non-coding transcripts out of the recently identified 400 candidate sRNAs [60] changed their levels in the absence of Hfq. Similarly, integrative analysis of Hfq regulon in *P. fluorescence* identified only four ncRNAs out of 87 whose abundance was dependent on Hfq [30]. Thus, in the light of observed extensive changes in transcriptomic and proteomic profiles observed in *B. pertussis*, the relatively small impact on sRNA levels suggests that Hfq exerts some of its regulatory activities in the sRNA-independent fashion or does not substantially contribute to sRNA stability in *B. pertussis*.

Collectively, this study reveals that impact of Hfq on the gene and protein expression profiles in *B. pertussis* is very profound. The Hfq regulon is comprised of hundreds of genes/proteins making almost 20 % of its genome and covering broad variety of genes and their products involved in different cellular processes. Obviously, these pleiotropic effects associated with loss of Hfq in *B. pertussis* cannot be completely ascribed to its role in posttranscriptional circuits but instead may be related to other global regulators that are themselves targets of Hfq regulation such as transcriptional factors. We assume that several observed effects are linked to impaired growth of the mutant. Especially increased production of proteins implicated in transport of metabolites and essential elements seems to compensate for the physiological defect introduced by deletion of the *hfq* gene. Finally, our study corroborated and further clarified the necessity of Hfq for physiological fitness and pathogenicity of *B. pertussis*. It will be of our primary interest to characterize the exact mechanism rendering the production and secretion of T3SS components strongly dependent on Hfq. Furthermore, we are currently characterizing function of several identified Hfq-dependent sRNAs in the physiology of *B. pertussis*.

## 4. Materials and Methods

### 4.1. Bacterial Strains and Growth Conditions

The *Bordetella pertussis* Tohama I strain [61] and its isogenic *hfq* deletion mutant were grown on Bordet-Gengou agar (BGA) plates supplemented with 15% sheep blood for 3 to 4 days at 37 °C. For liquid cultures, bacteria were grown in Stainer–Scholte (SS) medium [62] supplemented with 0.1% cyclodextrin and 0.5% casamino acids (Difco) at 37 °C. To harvest samples for RNA and protein isolation, the *B. pertussis* cells were grown overnight in SS medium to mid exponential phase of growth (OD_600_ ≈ 1.0). Three independent cultivation experiments were performed to collect three biological replicates for each of both strains for RNA and protein isolation.

### 4.2. RNA Isolation

Total RNA was isolated using TRI Reagent (Sigma, Darmstadt, Germany) according to manufacturer’s protocol. Removal of DNA was achieved by treatment of samples with TURBO DNA-free kit (Thermo Fisher Scientific). RNA quality and quantity was determined by agarose gel electrophoresis and using the Nanodrop 2000 machine (Thermo, Carlsbad, CA, USA). Furthermore, the RNA quality was assessed at sequencing facility (Vienna Biocenter Core Facility, NGS unit) on an Agilent 2100 Bioanalyzer device. All samples displayed RNA integrity numbers higher than 9.

### 4.3. Library Preparation and Deep Sequencing

Ribosomal RNA was depleted with the Ribo-Zero rRNA Removal Kit for Bacteria (Illumina, San Diego, CA, USA). Libraries were prepared using NEBNext^®^ Ultra™ II DNA Library Prep Kit for Illumina and sequenced on an Illumina HiSeq 2500 platform using HiSeqV4 chemistry with single-end 50-base-pair reads at the Vienna Biocenter Core Facilities Next Generation Sequencing unit. Reads were demultiplexed and quality trimming and adapter removal from the reads was performed using trimmomatic [63]. After quality control and adapter clipping, the reads were mapped to *B. pertussis* Tohama I reference genome using segemehl [64] with default parameters. Reads per gene counts were deduced with htseq-count with default parameters [65]. Differential gene expression analysis was performed with DESeq2 [66]. Genes with a |log_2_ fold change| > 1 and a *q*-value (*p*-value adjusted for multiple testing correction by the method of Benjamini and Hochberg [67]) < 0.05 were considered as significantly deregulated. RNA-seq data from the sequencing runs were deposited at the European Nucleotide Archive (ENA) under project accession number PRJEB32623.

### 4.4. Protein Isolation and Sample Preparation for Proteomics

Cultures of *B. pertussis* were pelleted by centrifugation (10,000× *g*, 4 °C, 10 min) to separate cell pellets and culture supernatants. Cells were resuspended in TEAB digestion buffer (100 mM Triethylammonium bicarbonate, pH 8.5, 2% sodium deoxycholate) and lysed by sonication. For analysis of supernatant fractions, supernatants were filtered through 0.22-μm filters and precipitated with 10% (*w*/*v*) trichloracetic acid (Sigma) overnight at 4 °C. Precipitated proteins were collected by centrifugation (14,000× *g*, 4 °C, 20 min), washed with 80% acetone (*w*/*v*) and finally dissolved in TEAB digestion buffer. Protein concentrations were determined using BCA protein assay kit (Thermo Fischer Scientific) and 20 µg of protein per sample were used for protein analysis. Cysteines were reduced with M Tris(2-carboxyethyl)phosphine (60 °C for 60 min) and blocked with 1M methyl methanethiosulfonate (10 min, room temperature). Samples were digested with trypsin (trypsin to protein ratio 1:20) at 37 °C overnight. Digestion of samples was stopped by addition of trifluoracetic acid (Sigma) to a final concentration of 1% (*v*/*v*). SDC was removed by extraction with ethylacetate [68] and peptides were desalted on C18 column (Michrom Bio, Auburn, CA, USA).

### 4.5. Label-Free Proteomic Analysis by LC-MS/MS

A nanoreversed phase column (EASY-Spray column, 50 cm × 75 µm ID, PepMap C18, 2 µm particles, 100 Å pore size) was used for LC-MS analysis. Mobile phase buffer A was composed of water and 0.1% formic acid. Mobile phase B was composed of acetonitrile and 0.1% formic acid. Samples were loaded onto the trap column (Acclaim PepMap300, C18, 5 µm, 300 Å wide pore, 300 µm × 5 mm) at a flow rate of 15 μL/min. Loading buffer was composed of water, 2% acetonitrile, and 0.1% trifluoroacetic acid. Peptides were eluted with gradient of B phase ranging from 4% to 35% over 60 min at a flow rate of 300 nL/min. Eluting peptide cations were converted to gas-phase ions by electrospray ionization and analyzed on a Thermo Orbitrap Fusion (Q-OT-qIT, Thermo Fischer). Survey scans of peptide precursors from 350 to 1400 m/z were performed at 12 resolution (at 200 *m*/*z*) with a 5 × 10^5^ ion count target. Tandem MS (MS^2^) was performed by isolation within 1.5-Th window with the quadrupole, HCD fragmentation with normalized collision energy of 30, and rapid scan MS analysis in the ion trap. The MS^2^ ion count target value was set to 10^4^ and the maximal injection time was 35 ms. Only those precursors with charge state 2–6 were sampled for MS^2^. The dynamic exclusion duration was set to 45 s with a 10 ppm tolerance around the selected precursor and its isotopes. Monoisotopic precursor selection was turned on. The instrument was run in top speed mode with 2 s cycles [69].

Raw data were imported into MaxQuant software (version 1.5.3.8) [70] for identification and label-free quantification of proteins. The false discovery rate (FDR) was set to 1% for peptides and minimum specific length of seven amino acids. The Andromeda search engine [71] was used for the MS/MS spectra search against the Uniprot *Bordetella pertussis* database (downloaded on November 2016), containing 3258 entries. Enzyme specificity was set as C-terminal to Arg and Lys, also allowing cleavage at proline bonds and a maximum of two missed cleavages. Dithiomethylation of cysteine was selected as fixed modification and N- terminal protein acetylation and methionine oxidation as variable modifications. The “match between runs” feature of MaxQuant was used to transfer identifications to other LC-MS/MS runs based on their masses and retention time (maximum deviation 0.7 min) and this was also used in quantification experiments. Protein abundance was calculated from obtained label-free protein intensities using the MaxLFQ algorithm described recently [72]. Proteins with less than four MS/MS spectral counts were removed from the analysis. Statistics and data interpretation were performed using Perseus 1.6.1.3 software [73]. The normalized label free intensities were compared between wt and *hfq* mutant and each abundance ratio was tested for significance with two-group *t*-test (*p*-value < 0.05). The *p*-values were further adjusted for multiple testing correction to control the false discovery rate at cut off of 0.05 using the permutation test (number of randomization 250). Proteins with corrected *p*-value (*q*-value) < 0.05 were considered as significantly modulated. For downstream analyses (e.g., GO term enrichment) only proteins which were detected by at least two unique peptides in at least two of the three biological replicates were considered. Proteins for which label free intensities were not obtained in any of the replicates of either the wt or the Δ*hfq* strain were considered as significantly modulated and defined as “ON/OFF”. The proteomics data were deposited to the ProteomeXchange Consortium via the PRIDE [74] partner repository with the dataset identifier PXD013953.

### 4.6. GO Term Enrichment Analysis

To gain a comprehensive functional annotation of the reference genome, gene ontology (GO) terms per gene were deduced using blast2go [75]. For the GO term enrichment analysis significantly deregulated genes from the transcriptome and proteome analysis were split into up- and downregulated genes and each gene set was analyzed separately. Each GO term which is associated with more than one gene in the gene set was tested for enrichment in the gene set compared to the whole transcriptome, applying a Fisher’s exact test. Afterwards, determined *p*-values were corrected for multiple testing by the method of Benjamini and Hochberg [67]. Enriched GO terms were further summarized and visualized by Revigo [76].

### 4.7. Transcriptome–Proteome Correlation Analyses

To correlate the effect of hfq gene deletion on the transcript and protein abundance globally, the log_2_FC of all genes the products of which were reliably detected (see Chapter 4.5) by the label-free quantification were compared. To this end, the ‘lm’ function from R was used to fit a linear model between these two datasets. Since the relative errors in log_2_FC measurements can be expected to be higher for genes with higher *p*-value, each data point was weighted in the course of model fitting by 1 - *q*-value where *q*-value represents the geometric mean of the *q*-values of the proteome and the transcriptome analysis.

### 4.8. Serum Killing Assay

Overnight-grown bacterial cultures were diluted in SS medium to 5 × 10^6^ bacteria/ ml of culture and supplemented either with intact or heat-inactivated (56 °C, 30 min) 10% human serum (Sigma No). Cells were incubated in parallel in the presence of both type of sera for 60 min at 37 °C in orbital incubator. Then the bactericidal activity was terminated by addition of 10 mM EDTA, serial dilutions of bacterial samples were plated onto BG agar and colony-forming units (CFU) were counted to assess bacterial survival. Survival was calculated as a percentage of CFU obtained from cultures treated with intact serum compared to CFUs from cultures treated with heat-inactivated serum (control, 100% survival). Mann–Whitney test was applied to assay the statistical significance of observed differences in sensitivity to serum killing.

## Figures and Tables

**Table 1:** List of genes showing consistently increased RNA and protein abundance in the *hfq* mutant.

Gene	RNA-seq^1^	Proteome^1^	Secretome^1^	Function
BP1358	4.18	3.93	2.78	TTT transporter
BP1487	4.34	2.92	1.65	TRAP transporter
BP1489	3.48	2.06	ND	TRAP transporter
BP2066	2.64	ON	8.80	TTT transporter
BP2090	1.92	ON	4.40	ABC transporter
BP2271	5.05	9.75	3.98	lipoprotein
BP2352	3.58	4.50	1.90	TRAP transporter
BP2667	2.11	6.47	5.72	adhesin FhaS
BP2692	1.51	2.10	1.90	ABC transporter
BP3501	3.36	4.86	ON	TTT transporter
BP3783	1.50	1.62	1.05	pertussis toxin subunit A
BP3784	1.80	1.14	1.04	pertussis toxin subunit B
BP3785	1.47	1.57	*0.81*	pertussis toxin subunit D
BP3786	1.14	ND	1.60	pertussis toxin subunit E
BP3787	1.49	1.76	1.26	pertussis toxin subunit C

^1^ Log_2_FC values of Δ*hfq*/wt comparison are shown for RNA-seq and proteomic analyses. Values which did not pass the statistical significance are shown in italics. ND: not determined in both strains in the respective analysis. ON: protein was not detected in the wt strain within the corresponding fraction.

**Table 2:** List of T3SS genes showing consistently decreased RNA and protein abundance in the *hfq* mutant.

Gene	Name	RNA-seq^1^	Proteome^1^	Secretome^1^
BP0500	*bopC*	*−0.69*	−1.19	OFF
BP2248	*bscJ*	−1.58	−2.16	ND
BP2250	*bcr4*	−1.81	−2.75	OFF
BP2251	*bcrH2*	−1.58	−1.44	ND
BP2252	*bopB*	−1.89	−2.23	OFF
BP2253	*bopD*	−1.85	−1.83	−9.95
BP2254	*bcrH1*	−1.26	*−0.96*	OFF
BP2256	*bsp22*	−1.22	−1.76	-9.13
BP2257	*bopN*	−0.85	−1.71	OFF

^1^ Log_2_FC values of Δ*hfq*/wt comparison are shown for RNA-seq and proteomic analyses. Values that did not pass the statistical significance are shown in italics. ND: not determined in both strains in the respective analysis. OFF: protein was not detected in the Δ*hfq* strain within the corresponding fraction.

## Supplementary Materials

Supplementary materials can be found at https://www.mdpi.com/1422-0067/20/12/3073/s1.

## Abbreviations

ABC	ATP-binding cassette
BGA	Bordet-Gengou agar
FDR	False discovery rate
TEAB	Triethylammonium bicarbonate
EDTA	Ethylenediamine tetra-acetic acid
CFU	Colony forming unit
DE	Differential expression
TTT	Tripartite tricarboxylate transporters
TRAP	Tripartite ATP-independent periplasmic transporters
PCA	Principal component analysis
GO	Gene ontology
LFQ	Label-free quantification
T3SS	Type 3 secretion system
FC	Fold change
